# The role of postoperative radiotherapy (PORT) in combined small cell lung cancer (C-SCLC)

**DOI:** 10.18632/oncotarget.16885

**Published:** 2017-04-06

**Authors:** Yu Men, Yang Luo, Yirui Zhai, Jun Liang, Qinfu Feng, Dongfu Chen, Zefen Xiao, Zongmei Zhou, Zhouguang Hui, Luhua Wang

**Affiliations:** ^1^ Department of Radiation Oncology, National Cancer Center/Cancer Hospital, Chinese Academy of Medical Sciences and Peking Union Medical College, Beijing 100021, China; ^2^ Department of Medical Oncology, National Cancer Center/Cancer Hospital, Chinese Academy of Medical Sciences and Peking Union Medical College, Beijing 100021, China; ^3^ Department of VIP Medical Services & Department of Radiation Oncology, National Cancer Center/Cancer Hospital, Chinese Academy of Medical Sciences and Peking Union Medical College, Beijing 100021, China

**Keywords:** carcinoma, combined small cell lung, surgery, radiotherapy, survival

## Abstract

**Purpose:**

To explore the value of radiotherapy in C-SCLC patients, especially in those receiving a radical resection.

**Results:**

The differences of survivals between the postoperative radiotherapy (PORT) and non-PORT groups were not statistically significant. But analyzing the benefits in subgroups, PORT significantly improved OS (*p* = 0.015), DFS (*p* = 0.026), LRFS (*p* = 0.008) and DMFS (*p* = 0.030) in stage III patients. For the patients with N2 stage, all survivals of the PORT group were also statistically significantly higher than non-PORT group (*p* = 0.018, 0.032, 0.008, 0.042). Patients with more than 10% of metastatic lymph nodes could get a significant benefit survivals by receiving PORT (*p* = 0.033, 0.030, 0.025, 0.031). Having a systematic dissection of more than 17 lymph nodes was a subset which could get better OS and LRFS by receiving PORT (*p* = 0.045, 0.048).

**Methods:**

Between Jan. 2004 to Dec. 2012, fifty-five patients diagnosed as C-SCLC after complete surgical resection in our center were retrospectively analyzed. The overall survival (OS), disease free survival (DFS), loco-regional recurrence free survival (LRFS), and distant metastasis free survival (DMFS) were calculated by Kaplan-Meier method.

**Conclusions:**

PORT can significantly improve the survival of C-SCLC patients with resected pathological pN2 stage. For the patients with a large percent of metastatic lymph nodes, PORT can also improve survivals.

## INTRODUCTION

Combined small cell lung cancer (C-SCLC) is defined as small cell lung cancer (SCLC) combined with an additional component that consists of any of the histological types of non-small cell lung cancer (NSCLC). C-SCLC is quite an uncommon cancer possessing about 1–3% of all SCLCs [1–5].

The diagnosis of C-SCLC mainly depends on complete examination of pathological specimen after surgery. With the development of screening and surgical techniques, more and more patients with early lung cancer are diagnosed and have the opportunity to receive surgery and complete pathological examination as well, which led to the increase of diagnosed C-SCLC. Therefore, it is necessary to explore the value of postoperative radiotherapy (PORT).

PORT improves the treatment results in patients with pN+ SCLC, as well as those with pIIIA-N2 NSCLC. But PORT for C-SCLC is suboptimized although the treatment of C-SCLC mainly refers to the guideline of SCLC. Furthermore, due to the low incidence and lack of attention, there is no study focusing on PORT for C-SCLC yet. Our single institutional study aimed to elucidate the effectiveness of PORT on survival of C-SCLC patients and to identify the subgroups which may most likely benefit from PORT.

## RESULTS

### Patient characteristics

Totally 55 consecutive patients were enrolled. Characteristics of the patients are presented in (Table 1). The median age of the whole group was 58 years (range, 35–76 years). Thirty patients (54.5%) had the component of squamous cell carcinoma (SCC). The T stage was T_1-2_ in 34 patients (61.8%) and T_3-4_ in 21 patients (38.2%). The N stage was N_0_ in 23 patients (41.8%), N_1_ in 14 patients (25.5%) and N_2_ in 18 patients (32.7%), respectively. In addition, stage III counted the most by 47.3%. Regardless of the sequence, most patients received chemotherapy (76.4%). Of all 55 patients, 14 (25.5%) received PORT. Twelve out of the 14 patients had detailed records of radiation and all received intensity modulated radiation therapy. The median total radiation dose was 60 Gy (range, 50Gy-66Gy). Seven patients received a total dose ≥60Gy and 5 patients less than 60Gy. The clinical characteristics were comparable between the PORT group and non-PORT group, except that there were more N_0-1_ patients and more patients receiving chemotherapy and less patients receiving PCI in the non-PORT group (as shown in Table 1).

**Table 1:** Patient characteristics (*N* = 55)

	All		PORT		Non-PORT		*p* value
	No.	%	No.	%	No.	%	
Age, years	58(35–76)		58 (42–73)		59 (35–76)		0.391
≤ 60	34	61.8	10	71.4	24	58.5	
> 60	21	38.2	4	28.6	17	41.5	
Sex							0.975
Male	47	85.5	12	85.7	35	85.4	
Female	8	14.5	2	14.3	6	14.6	
Component							0.397
SCC	30	54.5	9	64.3	21	51.2	
Non-SCC	25	45.5	5	35.7	20	48.8	
Stage, AJCC 7^th^							0.140
I-II	29	52.7	5	35.7	24	58.5	
III	26	47.3	9	64.3	17	41.5	
T stage, AJCC 7^th^							0.826
T_1-2_	34	61.8	9	64.3	25	61.0	
T_3-4_	21	38.2	5	35.7	16	39.0	
N stage, AJCC 7^th^							0.008*
N_0_	23	41.8	1	7.2	22	53.7	
N_1_	14	25.5	5	35.7	9	21.9	
N_2_	18	32.7	8	57.1	10	24.4	
Dissected lymph nodes(DLN)							0.702
≤ 17	29	52.7	8	57.1	21	51.2	
> 17	26	47.3	6	42.9	20	48.8	
Metastatic lymph nodes(MLN)							0.715
0–3	45	83.6	11	78.6	34	82.9	
≥ 4	10	16.4	3	21.4	7	17.1	
Metastatic lymph nodes(MLN)							0.960
≤ 10%	39	70.9	10	71.4	29	70.7	
> 10%	16	29.1	4	28.6	12	28.3	
Chemotherapy							0.016*
No	13	23.6	0	0.0	13	31.7	
Yes	42	76.4	14	100.0	28	68.3	
PCI							0.003*
No	50	91.9	10	71.4	40	97.6	
Yes	5	9.1	4	28.6	1	2.4	

### Survivals in the whole group

**Figure 1:**
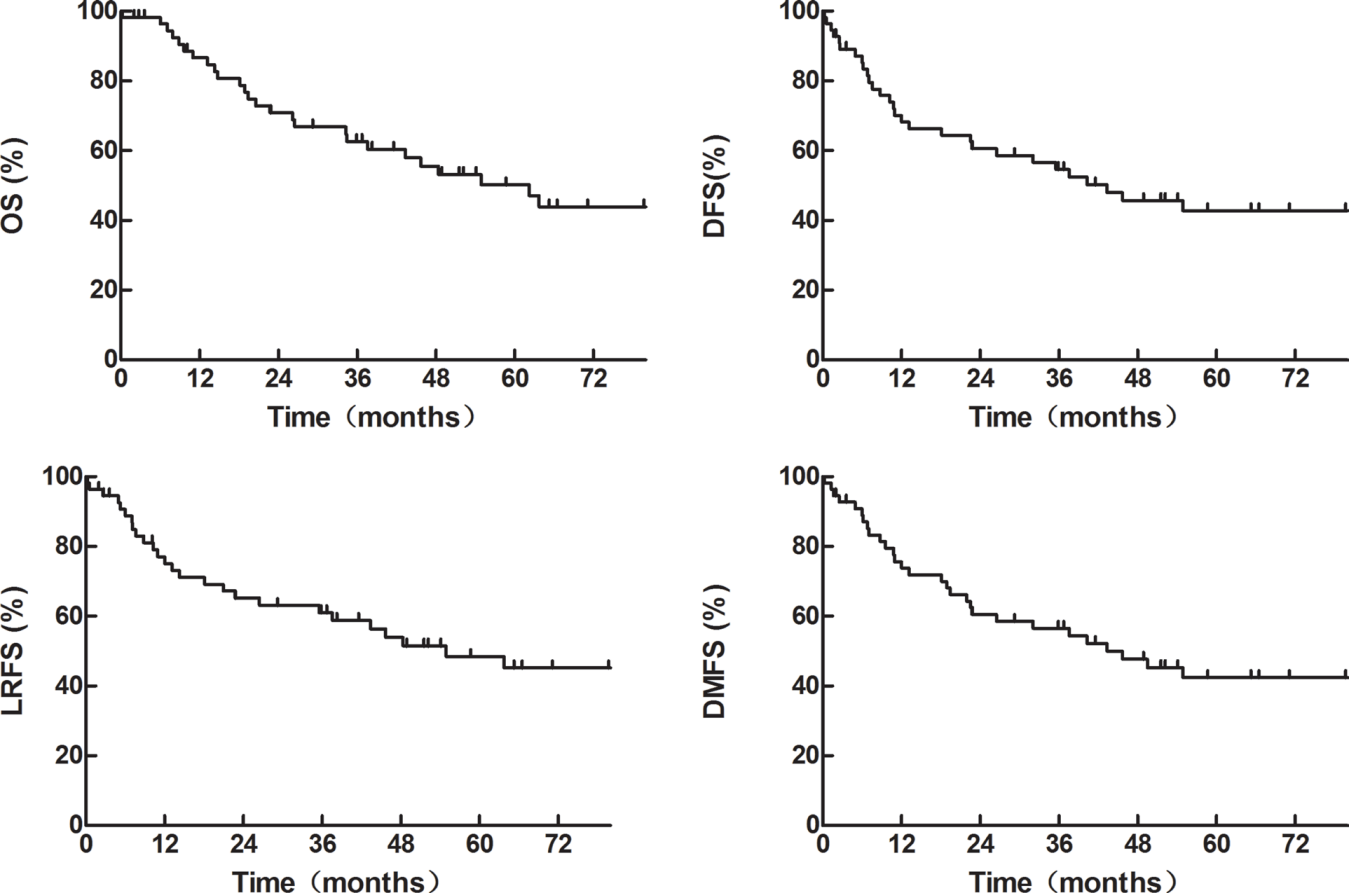
OS, DFS, LRFS and DMFS of the whole group of 55 patients

The median follow-up was 56.4 months (range, 2.0–127.2 months). The median OS, DFS, LRFS and DMFS were 62.2 months, 43.4 months, 54.9 months and 43.4 months, respectively. As shown in (Figure 1), the 1-, 3-, and 5-year OS rates were 86.6%, 62.6% and 50.2%, respectively, and the corresponding DFS rates were 70.1%, 54.6%, and 42.8%. The 1-, 3-, and 5-year LRFS rates were 77.1%, 61.1% and 48.4%, respectively, and the corresponding DMFS rates were 75.7%, 56.6%, and 42.5%.

### Effect of radiotherapy on survivals

**Figure 2:**
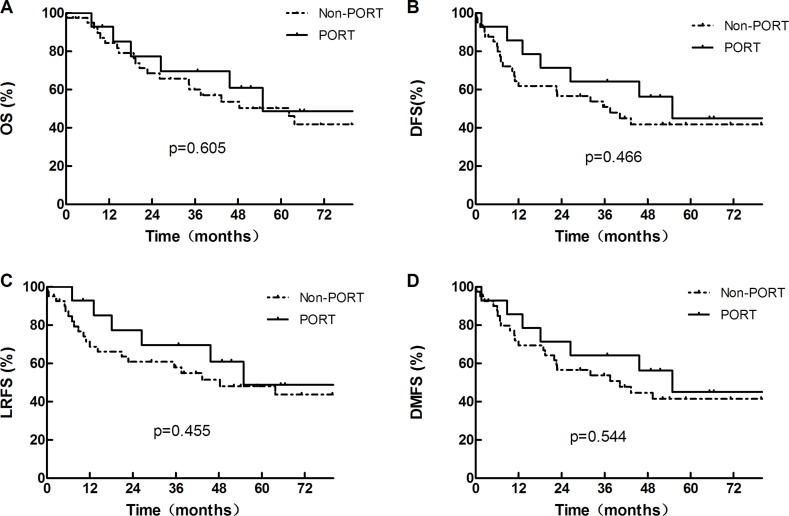
OS, DFS, LRFS and DMFS of the whole group of 55 patients, divided according to whether or not receiving PORT

For patients in the PORT and non-PORT groups, the median OS were 54.9 months and 62.2 months, respectively (χ^2^ = 0.267, *p* = 0.605) (Figure 2A). The 1-, 3- and 5-year OS rates were 92.9%, 69.6% and 48.8% in the PORT group, and 84.4%, 60.1% and 50.4% in the non-PORT group, respectively. The 1-, 3- and 5-year DFS rates were 85.7%, 64.3% and 45.0% in the PORT group, and 64.5%, 51.1% and 41.8% in the non-PORT group, respectively (χ^2^ = 0.532, *p* = 0.466) (Figure 2B). The 1-, 3-, and 5-year LRFS rates were 92.9%, 69.6%, and 48.8% in the PORT group and 71.4%, 58.0%, and 48.1% in the non-PORT group, respectively (χ^2^ = 0.558, *p* = 0.455) (Figure 2C). The 1-, 3- and 5-year DMFS rates were 85.7%, 64.3% and 45.0% in the PORT group, and 72.0%, 53.8% and 41.4% in the non-PORT group, respectively (χ^2^ = 0.367, *p* = 0.544) (Figure 2D). Though the OS, DFS, LRFS and DMFS all trended to be better in the PORT group than in non-PORT group, the differences were not statistically significant. On multivariate analysis, PORT was not significant positive prognostic factors for OS (HR = 0.516, 95%CI 0.194–1.371, *p* = 0.185).

### Subgroup analyses

Results of the subgroup analyses are shown in (Table 2). PORT significantly improved the survivals of patients with stage III or N_2_ disease, as well as those with more than 10% of metastatic lymph nodes. For the patients with stage III disease, the 1-, 3- and 5-year OS rates were 88.9%, 76.2% and 61.0% in the PORT group, which were statistically significantly higher than the corresponding rates of 67.2%, 20.2% and 13.4% in the non-PORT group (*p* = 0.015). The DFS, LRFS and DMFS in the PORT group were also significantly improved comparing with the non-PORT group (*p* = 0.026, 0.008 and 0.030, respectively). For patients with N_+_ disease, the OS, DFS, LRFS and DMFS in the PORT group were significantly higher than those in the non-PORT group (*p* = 0.012, 0.006, 0.003 and 0.010, respectively). However, when analyzed N_1_ and N_2_ separately, the significant difference only existed in N2 disease (*p* = 0.018, 0.032, 0.008 and 0.042, respectively) while the difference was marginally seen in N1 disease. Accordingly, patients with more than 10% of metastatic lymph nodes also had better survivals in the PORT group (*p* = 0.033, 0.030, 0.025 and 0.031, respectively). Moreover, Patients having more than 17 DLNs had a significantly better OS (*p* = 0.045) and LRFS (*p* = 0.048), but not better DFS (*p* = 0.109) or DMFS (*p* = 0.121) in the PORT group than the non-PORT group. PORT also markedly increased the survival rates in patients with T_3-4_ tumors or with ≥ 4 positive lymph nodes, but the difference was not statistically significant which may due to the small samples. On the contrary, PORT seemed to deteriorate the survivals in patients with T_1-2_ or I-II disease though the difference was not statistically significant. Whether or not having SCC was not a significant factor for PORT. As for the patients with smoking history, PORT did not influence the OS (*p* = 0.843), DFS (*p* = 0.901), LRFS (*p* = 0.945) and DMFS *p* = 0.987). In the patients who received chemotherapy, the PORT group had similar survivals to the non-PORT group (OS, *p* = 0.702; DFS, *p* = 0.681; LRFS, *p* = 0.508; and DMFS, *p* = 0.777).

**Table 2:** Effect of PORT on OS, DFS, LRFS and DMFS of patients with different factors

Factor	5y-OS (%)			5y-DFS (%)			5y-LRFS (%)			5y-DMFS (%)		
	PORT	Non- PORT	*p* value	PORT	Non- PORT	*p* value	PORT	Non- PORT	*p* value	PORT	Non- PORT	*p* value
Stage, AJCC 7th												
I-II	30.0	75.2	0.165	30.0	59.5	0.489	30.0	70.4	0.232	30.0	58.8	0.423
III	61.0	13.4	0.015*	53.3	13.8	0.026*	61.0	13.7	0.008*	53.3	13.6	0.030*
T stage, AJCC 7th												
T_1-2_	56.3	61.7	0.922	50.0	59.4	0.993	56.3	62.3	0.842	18.6	59.4	0.939
T_3-4_	30.0	31.3	0.498	30.0	14.8	0.318	30.0	23.7	0.362	30.0	14.7	0.405
N stage, AJCC 7th												
N_0_	0.0	83.8	0.115	0.0	64.9	0.457	0.0	78.6	0.201	0.0	64.2	0.468
N_+_	57.5	11.8	0.012*	52.7	12.1	0.006*	57.5	12.0	0.003*	52.7	11.9	0.010*
N_1_	40.0	12.7	0.313	40.0	13.0	0.135	40.0	12.7	0.183	40.0	13.0	0.188
N_2_	72.9	11.1	0.018*	62.5	11.4	0.032*	72.9	11.4	0.008*	62.5	11.1	0.042*
DLN												
≤ 17	16.7	49.9	0.248	16.7	43.3	0.599	16.7	50.5	0.410	16.7	43.3	0.519
> 17	100	51.2	0.045*	83.3	40.2	0.109	100	46.0	0.048*	83.3	39.3	0.121
MLN												
0-3	38.9	60.0	0.758	35.4	49.3	0.990	38.9	56.9	0.933	35.4	48.9	0.896
≥ 4	66.7	0.0	0.155	66.7	0.0	0.153	66.7	0.0	0.096	66.7	0.0	0.161
≤ 10%	37.0	69.4	0.332	33.3	55.9	0.549	37.0	66.0	0.474	50.0	55.5	0.454
> 10%	75.0	10.0	0.033*	75.0	0.0	0.030*	75.0	0.0	0.025*	75.0	0.0	0.031*
Component												
SCC	54.7	56.3	0.402	48.6	44.7	0.480	54.7	50.8	0.311	48.6	43.8	0.526
Non-SCC	40.0	45.0	0.704	40.0	39.4	0.924	40.0	45.3	0.913	40.0	39.4	0.990

## DISCUSSION

C-SCLC contains the component of both SCLC and NSCLC. However, there are different applications of PORT between SCLC and NSCLC. PORT improves the treatment results in patients with pN+ SCLC, but those with pIIIA-N2 in NSCLC. Therefore, it's a question that whether C-SCLC should follow the guideline of SCLC or NSCLC or having its own when confronting the selection of PORT, especially in current circumstances that more and more patients were diagnosed as C-SCLC after surgery.

The rationale behind PORT is to kill malignant cells remaining in tumor bed, at the resection margins, or in the adjacent lymph nodes after surgery and so to reduce local and regional recurrences and improve survival. At present, the selection of PORT in C-SCLC mainly depends on the experience of doctors, lacking of clinical evidences. In both SCLC and NSCLC, PORT has certain indications. If giving PORT indiscriminately, there are clear evidences showing no benefit, even detrimental effects for patients with completely resected lung cancer [6, 7]. The meta-analysis published in 1998, which contained 9 randomized controlled trials and 2128 NSCLC patients, concluded that PORT was detrimental (HR 1.21, *p* = 0.001): twenty-one percent increase in the relative risk of death and 2-year survival rates of 48% for PORT and 55% for non-PORT group [6]. The updated results were largely unchanged [8]. Liu *et al*. [7] revealed that, in SCLC, the OS in the whole PORT group comparing with the non-PORT group was not significantly increased (*p* = 0.26). Consistently, our results show that for whole group, the survival rates are not different in the PORT group and non-PORT group. On multivariate analysis, PORT was also not significant prognostic factors. Therefore, recognizing the subgroups that could benefit from PORT is more meaningful.

In the treatment of SCLC, PORT is recommended to the patients with positive lymph nodes through the detection of surgical specimens. Liu *et al*. [7] revealed that PORT significantly reduced local-regional recurrence (LRR) and improved OS in patients with regional metastasis SCLC. In patients with N1 disease, the median OS were 40 months in the PORT group versus 14 months in the non-PORT group (*p* = 0.032). The corresponding OS in N2 patients were 35 months versus 17 months, respectively (*p* = 0.040). For patients with N1 disease, the 3-year LRR rate was 0.0% in the PORT group versus 14.3% in the non-PORT group (*p* = 0.037). The corresponding LRR rate in N2 patients was 4.2% versus 56.6% (*p* < 0.001). For NSCLC, the subset analysis of meta-analysis published in 1998 indicated that in patients with stage III or pN_2_, survival was slightly better with PORT [6]. In 2006, a retrospective analysis of the SEERs database including 7,465 patients also showed an increase in overall survival in pN_2_ disease (HR = 0.855, *p* = 0.0077) [9]. Dai et al. [10] presented that PORT could significantly improve the survival of patients with resected pathological stage IIIA–N_2_ NSCLC. Recently, Patel et al. [11] conducted a meta-analysis to evaluate the role of PORT based on the use of linear accelerators in pN_2_ lung cancer. The OS and LRFS of PORT group had both significantly improved (HR 0.77, *p* = 0.020; HR 0.51, *p* < 0.001, respectively). In our study, similarly as SCLC and NSCLC,, PORT could significantly improve OS, DFS, LRFS and DMFS of C-SCLC patients with stage III or pN2 stage. For these stages of C-SCLC, PORT may be recommended. Nowadays, the treatment of C-SCLC mainly refers to the guideline of SCLC. However, unlike SCLC recommending PORT in N1 disease, C-SCLC with N1 disease could not get benefit from PORT, which may due to the component of NSCLC. In the other hand, PORT didn't influence the survival of patients with component of SCC or with non-SCC. This indicates that, although the component of NSCLC may affect the use of PORT, the specific kind of NSCLC makes no difference.

The survival rates of patients with a large ratio of involved lymph nodes were significantly improved when receiving PORT. It has been reported that the number of metastatic mediastinal lymph nodes was a significant prognostic factor. Local and distant control are decreased with the increase of positive hilar or mediastinal lymph nodes [12–14]. Other than the number of MLN, the ratio of MLN or the lymph node ratio (LNR) has been proposed as a more useful prognostic metric because of its incorporation of both the number of positive nodes and the total number of examined nodes. Damien et al. [15] analyzed the benefit of PORT by nodal stage and LNR. It turned out that a high LNR was associated with a poorer survival in resected node-positive NSCLC. This may be the reason why PORT could improve survival of these patients.

PORT may have an important position in the treatment of patients having more DLN. The frequency of distant metastases is still very high after the resection of primary tumor and cleaning of regional lymph nodes. Even more, the rate of distant metastases is significantly higher than the incidence of local failure. Therefore, a survival benefit of PORT could only be acquired if microscopic metastatic disease is also effectively controlled. The more lymph nodes being excised, the less possibility of remaining disease. Thus the benefits obtained by using PORT can bring out.

As a retrospective analysis, our study has some limitations. First, all of the patients came from our single institution and the number of cases is limited. The results should be interpreted cautiously as selection bias may exist. Second, the median dose of RT in our data was 60 Gy, which is higher than the recommended dose of PORT for lung cancer and may cause an excess radiation pneumonopathy or cardiac disease. Third, the proportion ratio of each component of C-SCLC may have prognostic significance and play an important role in the selection of therapies. However, the information of our pathologic diagnosis is not detailed enough to support the correspondent analysis.

## MATERIALS AND METHODS

### Study population

From Jan. 2004 to Dec. 2012, consecutive patients with pathologically diagnosed C-SCLC after complete surgery were enrolled. Patients with positive surgical margin were excluded. Initial evaluations included physical and hematological examination, chest CT scans or PET-CT, bronchoscopy, ultrasound, brain MRI and bone scan. Pathological diagnosis was based on specimens from surgery. C-SCLC was staged according to the 7th edition of the American Joint Committee on Cancer (AJCC) tumor-node-metastasis (TNM) classification system. The medical records and follow-up data of the patients were retrospectively analyzed.

### Surgery

All patients underwent surgery of curative purpose. The types of surgical resection included wedge resection, sleeve resection, lobectomy and pneumonectomy.

### Radiotherapy

The administration of radiation therapy was based on the attending radiation oncologist's decision and partially the surgeon's suggestion. The techniques of PORT included three-dimensional conformal radiotherapy (3D-CRT) and intensity modulated radiotherapy (IMRT). The clinical target volume (CTV) included the tumor bed, subcarinal nodes, ipsilateral mediastinum and ipsilateral hilum. The planning target volume (PTV) was defined as CTV plus 0.5 cm margins. PORT was administered with linear accelerator using 6 to 8 MV X-rays at 1.8Gy-2.0Gy per fraction, 5 days per week, to a total prescription dose of 60 Gy.

### Outcome measures

Patients were followed-up every 3 months for the first year, then every 3 to 6 months thereafter. Overall survival (OS) was defined as the time between initial treatment and death or the last follow-up. Disease-free survival (DFS) was defined as the time between initial treatment and the relapse of disease or death or the last follow-up. Loco-regional recurrence-free survival (LRFS) was defined as the time between initial treatment and the recurrence of the primary tumor or regional lymph node, death or the last follow-up. Distant metastasis-free survival (DMFS) was measured from the date of initial treatment to the date of distant metastasis, death or the last follow-up.

### Statistical analysis

The Kaplan-Meier method was used to estimate OS, DFS, LRFS and DMFS. The difference of survival between patients with different factors was compared using the log-rank test. Statistically significant difference was set as *p* < 0.05.

## CONCLUSIONS

For pathological N_2_ stage C-SCLC patients, PORT can significantly improve OS, DFS, LRFS and DMFS. Moreover, the patients with a large percent of metastatic lymph nodes also have significant beneficial survivals. In addition, the PORT group has higher OS and LRFS than the control when patients had a large number of DLN through surgery. Large-scale and multi-institution studies are needed to further evaluate the role of PORT in C-SCLC.
